# Cytogenetic and molecular diagnosis of Fanconi anemia revealed two hidden phenotypes: Disorder of sex development and cerebro‐oculo‐facio‐skeletal syndrome

**DOI:** 10.1002/mgg3.694

**Published:** 2019-05-23

**Authors:** Abir Ben Haj Ali, Ahlem Amouri, Marwa Sayeb, Saloua Makni, Wajih Hammami, Chokri Naouali, Hamza Dallali, Lilia Romdhane, Anu Bashamboo, Kenneth McElreavey, Sonia Abdelhak, Olfa Messaoud

**Affiliations:** ^1^ Laboratory of Histology and Cytogenetics Institut Pasteur de Tunis, University Tunis El Manar Tunis Tunisia; ^2^ Laboratory of Biomedical Genomics and Oncogenetics Institut Pasteur de Tunis, University Tunis El Manar Tunis Tunisia; ^3^ Children's Hospital of Tunis Tunis Tunisia; ^4^ Human Developmental Genetics Institut Pasteur de Paris Paris France

**Keywords:** autozygosity mapping, Comorbidity, genetic counseling, incidental findings, whole exome sequencing

## Abstract

**Background:**

Several studies have shown a high rate of consanguinity and endogamy in North African populations. As a result, the frequency of autosomal recessive diseases is relatively high in the region with the co‐occurrence of two or more diseases.

**Methods:**

We report here on a consanguineous Libyan family whose child was initially diagnosed as presenting Fanconi anemia (FA) with uncommon skeletal deformities. The chromosome breakage test has been performed using mitomycin C (MMC) while molecular analysis was performed by a combined approach of linkage analysis and whole exome sequencing.

**Results:**

Cytogenetic analyses showed that the karyotype of the female patient is 46,XY suggesting the diagnosis of a disorder of sex development (DSD). By looking at the genetic etiology of FA and DSD, we have identified p.[Arg798*];[Arg798*] mutation in *FANCJ* (OMIM #605882) gene responsible for FA and p.[Arg108*];[Arg1497Trp] in *EFCAB6* (Gene #64800) gene responsible for DSD. In addition, we have incidentally discovered a novel mutation p.[Gly1372Arg];[Gly1372Arg] in the *ERCC6* (*CSB*) (OMIM #609413) gene responsible for COFS that might explain the atypical severe skeletal deformities.

**Conclusion:**

The co‐occurrence of clinical and overlapping genetic heterogeneous entities should be taken into consideration for better molecular and genetic counseling.

## 1 INTRODUCTION

North African populations are characterized by their heterogeneous ethnic background and high rate of inbreeding. The consanguinity rate is influenced by several factors including demographic, religious, cultural, and socioeconomic conditions (Romdhane & Abdelhak, 2011). The high rate of consanguinity has been usually associated with the emergence of autosomal recessive diseases at high frequencies (Teebi, 1994). In fact, it was reported that among 346 genetic disorders described in Tunisia, 62.9% are autosomal recessive (Romdhane & Abdelhak, 2011). Fanconi anemia (FA) is among the diseases for which incidence is increased by consanguinity (Hadiji et al., 2012), hence allowing founder ancestral mutations such as exon 15 deletion in *FANCA* (OMIM #607139) gene to be frequently observed (Amouri et al., 2014). Indeed, more than 142 patients with a consanguinity rate of 86% have been registered in the Tunisian Fanconi Anemia Registry (TFAR) (Hadiji et al., 2012). As the Tunisian population structure is very similar to that of neighboring countries, FA prevalence is expected to be just as high in these countries. In Egypt, among 48 patients clinically suspected to have FA, the diagnosis has been cytogenetically confirmed for 31 cases for whom the consanguinity rate reaches 97% (Temtamy et al., 2007). Jewish FA patients have been reported mostly from Morocco and Tunisia (Tamary et al., 2000).In Libya, the consanguinity rate reaches 37.6% in Benghazi (Abudejaja et al., 1987). Unfortunately, epidemiological studies for numerous cases including FA are lacking due to an unstructured health system.

FA is a rare genetic disease characterized by variable congenital defects, hematological problems, and cancer. A large clinical heterogeneity is associated with this syndrome. Indeed, the clinical manifestation differs from one patient to another and it is due to several factors that remain unknown. As the biological diagnosis of FA relies on double‐stranded DNA breaks in the chromosomes of FA patients when cultured with DNA interstrand cross‐linking agents, such as diepoxybutane (DEB) (Auerbach, 2003) or mitomycin C (MMC) (Cervenka, Arthur, & Yasis, 1981), all FA genes are known to play a role in double‐stranded DNA (dsDNA) breaks repair. In the absence of a clear phenotype–genotype correlation, molecular diagnosis of FA is complicated. Until now, 21 responsible FA genes have been defined (Mamrak, Shimamura, & Howlett, 2017). The gravity of this disease justifies an adequate genetic counselling and prenatal or preimplantation genetic diagnosis, which requires precise identification of the pathogenic mutations.

Here, we report on a Libyan FA child who was addressed with a suspicion of FA but further investigations showed co‐occurrence of three different rare genetic and clinical entities.

## 2 PATIENTS AND METHODS

### 2.1 Ethical compliance

This study was conducted in accordance with the declaration of Helsinki and the ethical standards of the Institut Pasteur de Tunis Institutional Review Board (Registration number IRB00005445, FWA00010074). Informed consent of the legal representatives of the patient was obtained.

### 2.2 Clinical and genealogical description

**Figure 1:**
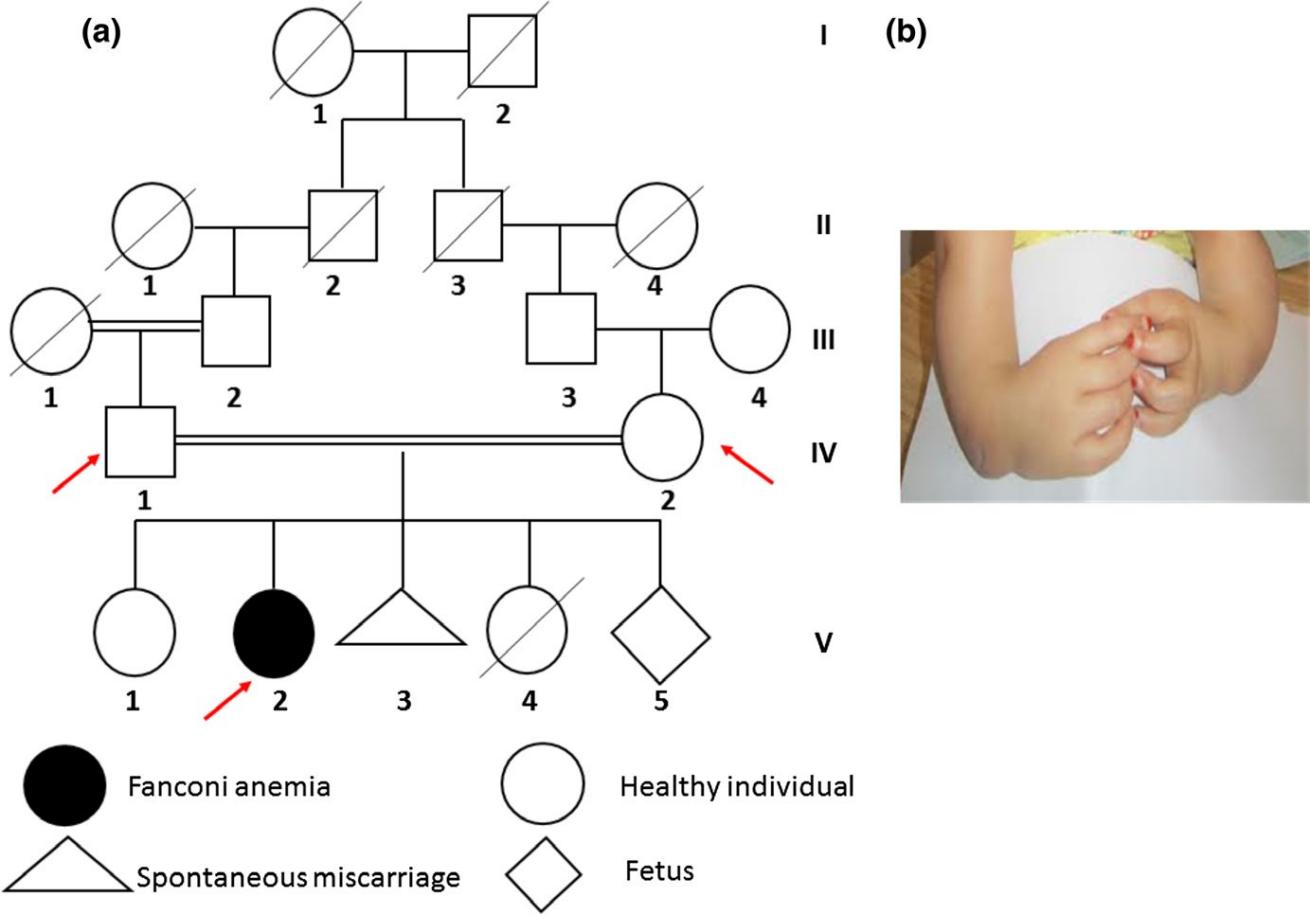
(a) Pedigree of the Libyan FA family; arrows indicate the members with available DNA. (b) Phenotypical features of S156‐V‐2 patient

A female patient S156‐V‐2, aged 4 years and born to young healthy second‐degree cousins was addressed to our department in Institut Pasteur de Tunis (IPT) for FA cytogenetic test (Figure1a). Physical examination revealed: growth retardation, microcephaly, facial dysmorphism, beaked nose, thin lips, upper lip overhanging the lower one, café au lait spots, and severe skeletal deformities including bilateral radial agenesis with absent thumbs (Figure1b). The eldest sister V‐1 was healthy. The mother had a history of two additional pregnancies, the first resulted in a spontaneous miscarriage while the second was marked by aneonatal death due to congenital malformations mainly marked by severe polydactyly; however, no cytogenetic nor molecular studies have been performed.

### 2.3 Chromosomal breakage test

A standard chromosomal breakage test with MMC in peripheral blood cultures has been conducted to assess chromosomal hypersensitivity to clastogenic agents as previously detailed (Talmoudi et al., 2013).

### 2.4 Genetic analysis

After obtaining a written consent from the parents, EDTA‐blood samples were received and genomic DNA was extracted applying the standard salting‐out method (Miller, Dykes, & Polesky, 1988). Whole exome sequencing (WES) was performed using 5 μg of the genomic DNA sample. Sanger sequencing was carried out to confirm the mutations identified by WES. All PCR products were directly sequenced on an ABI 3,130 automated sequencing system (Applied Biosystems, CA). Sequences were then compared to published sequences of the *FANCJ*gene (NM_032043.2), *EFCAB6* gene (NM_022785.4), and *ERCC6* gene (NM_000124.4) using the Sequencher 5.0 software program package (Gene Codes, MI, and USA).

#### 2.4.1 Prescreening of exon 15 deletion in *FANCA* gene

The *FANCA* gene is the most frequent gene being responsible of almost 90% of FA in Tunisian (Bouchlaka et al., 2003), Algerian, and Libyan patients (unpublished data); thus the patient reported here was initially screened using a PCR‐based approach for exon 15 deletion in *FANCA* gene which is a common founder mutation in Southern Tunisia (Amouri et al., 2014) and among patients from Libya and Algeria (unpublished data).

#### 2.4.2 Homozygosity mapping

Taking into account the recessive trait of FA and the consanguinity of the index case, we applied homozygosity mapping strategy to look for regions of homozygosity by descent and consequently locate the responsible gene(s) with the causative mutation(s). Whole exome Single Nucleotide Polymorphism (SNP) genotype data from the index case and her parents were analyzed for the presence of runs of homozygosity (McQuillan et al., 2008) through the Runs of Homozygosity (ROH) tool from PLINK v1.06 (http:// pngu.mgh.harvard.edu/purcell/plink). We kept the default settings of the “homozygous” command. Then, we applied the FSuite software for better visualization and determination of these ROHs (Gazal, 2014).

#### 2.4.3 Whole exome sequencing

In order to define disease‐relevant variants, we realized WES for the index case S156‐V‐2 and her parents. WES and bioinformatics' analysis were performed by Oxford Gene Technology (OGT; London, UK). Briefly, samples were prepared according to Agilent's SureSelect Protocol Version 1.2 (Agilent Technologies; Santa Clara, CA). Obtained libraries were pooled prior to sequencing with each sample at a final concentration of 10nM. Paired‐end sequencing was performed on the Illumina HiSeq2000 platform using TruSeq v3 chemistry. Read files (Fastq format) were generated from the sequencing platform via the manufacturer's proprietary software. Burrows‐Wheeler Aligner's sample algorithm, for paired‐end–short read alignment (BWA‐short version 0.6.2) (Li & Durbin., 2009), was used for mapping to the human genome (hg19/b37). Sequence alignment Map (SAM) was converted to Binary Alignment Map (BAM) using Sam tools version 0.1.18. (Li et al., 2009). Further BAM files processing was performed using Picard tools version 1.89 (http://broadinstitute.github.io/picard) and Genome Analysis Tool Kit (GATK) version 1.6 (https://software.broadinstitute.org/gatk/documentation/version-history.php)(McKenna et al., 2010), which was also used for variant calling and variant filtering. Single nucleotide polymorphism (SNP) novelty was determined against dbSNP137. Candidate variants were analyzed by a range of web‐based bioinformatic tools like the EnsEMBL SNP Effect Predictor (https://www.ensembl.org/info/docs/tools/vep/index.html) (McLaren et al., 2016) and VarioWatch (http://grch37.genepipe.ncgm.sinica.edu.tw/variowatch/main.do) (Cheng et al., 2012).All variants were screened against the Human Gene Mutation Database Public version (Biobase) (http://www.hgmd.cf.ac.uk/ac/index.php) (Stenson et al., 2003). In silico analysis was done to evaluate the likely pathogenicity of variants using PolyPhen (http://genetics.bwh.harvard.edu/pph2/) (Sunyaev et al., 2001), SIFT (https://sift.bii.a-star.edu.sg/) (Ng & Henikoff, 2003), Align‐GVGD(http://agvgd.iarc.fr) (Tavtigian et al., 2006), Pmut (http://mmb2.pcb.ub.es/PMut/) (Ferrer‐Costa et al., 2005), and Fruitfly (http://www.fruitfly.org/seq_tools/splice.html) (Reese, Eeckman, Kulp, & Haussler, 1997) for splice‐site predictions.

## 3 RESULTS

**Figure 2:**
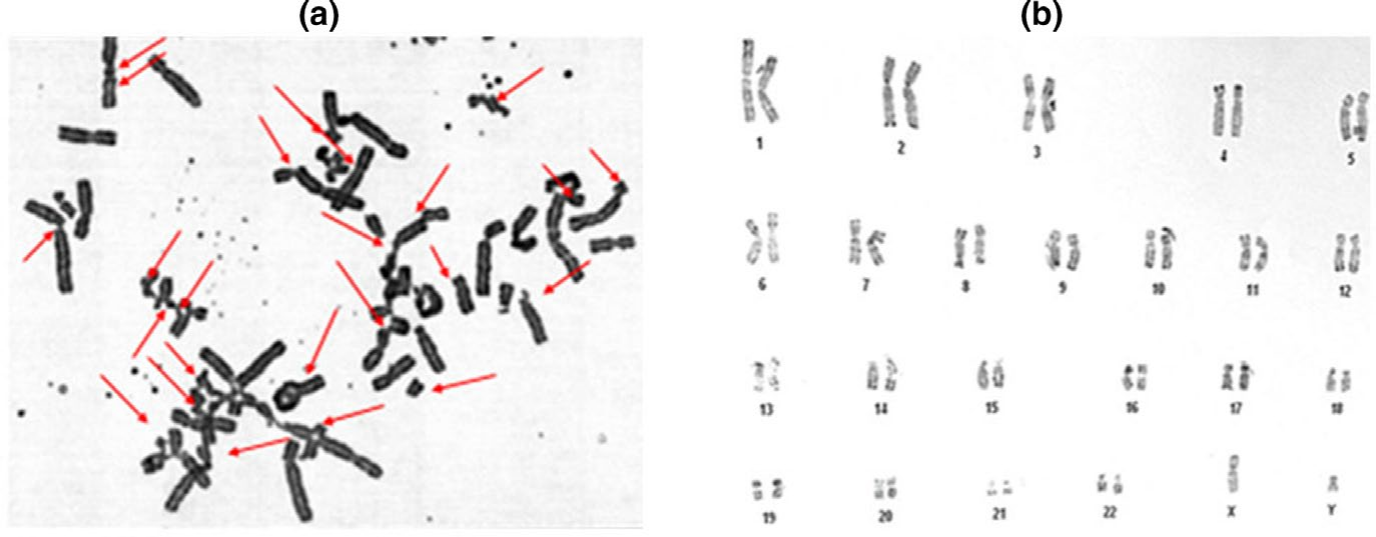
(a) Metaphase spread from the patient S156‐V‐2, after exposure to Mitomycin C (MMC), exhibiting multiple chromosomal breaks and radial formations that are indicated by arrows. (b) Constitutional karyotype 46,XY of the patient S156‐V‐2

In our study, we investigated at clinical, cytogenetic, and molecular levels a patient with a suspicion of FA. The cytogenetic instability assessment revealed a high frequency of chromosomal breakages (8.64 breaks/cell) compared to control (0.062 breaks/cell) (Figure 2a). In addition to clinical features, these results confirmed the diagnosis of FA. The karyotype analysis and the standard chromosomal breakage test showed 46,XY in all analyzed mitoses. The father IV‐1 and the mother IV‐2 had both a normal karyotype (46,XY; 46,XX, respectively). This incidental discovery is suggesting sex reversion (Figure 2b).

**Figure 3:**
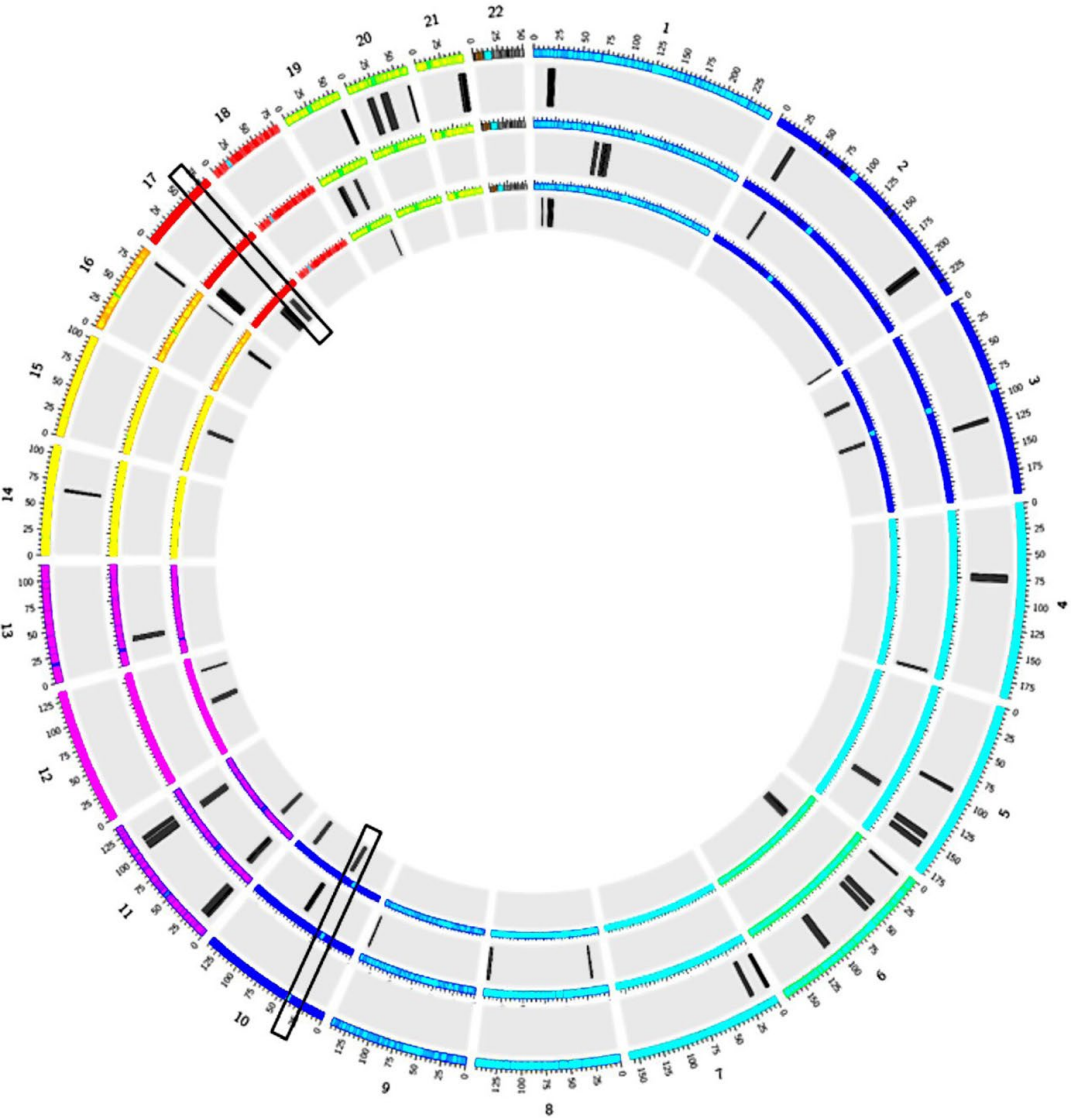
Circular representation of autosomal chromosomes indicating regions of homozygosity (ROH) drawn from WES data of the Libyan family using FSuite software (from outside to inside: IV‐1, IV‐2 and S156‐V‐2). Overlapping of the father's, the mother's, and the patient's ROH map showed that only two ROH (in rectangular boxes) are identical by descent

**Figure 4:**
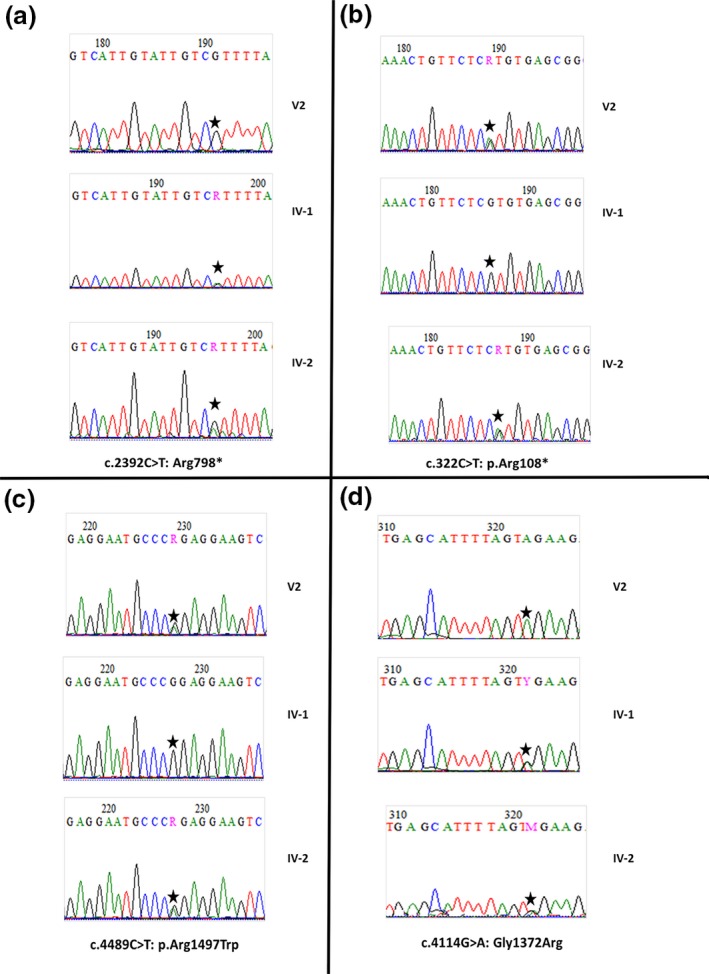
Sequencing results of the proband S156‐V‐2 and her parents (IV‐1, IV‐2): (a) a nonsense mutation c.[2392C> ];[2392C>T]: p.[Arg798*];[Arg798*] in exon 17 of *BRIP1/FANCJ*; (b) a nonsense mutation c.[322C>T]: p.[Arg108*] in exon 4 of *EFCAB6* gene; (c) a missense mutation c.[4489C>T]: p.[Arg1497Trp]in exon 32 of *EFCAB6* gene and (d) a missense mutation c.[4114G>A];[4114G>A]: p.[Gly1372Arg];[Gly1372Arg] in exon 21 of *ERCC6* gene

After excluding exon 15 deletion in *FANCA* gene, WES data analysis allowed identification of a nonsense mutation c.[2392C>T];[2392C>T]: p.[Arg798*];[Arg798*] at homozygous state in exon 17 of *BRIP1/FANCJ* gene which is responsible for FA. Homozygosity mapping showed the presence of two IBD‐ROH (Identity by descent‐Runs of homozygosity) (Figure3). The first one is located in chromosome 17 and contains the *BRIP1/FANCJ* gene, thus supporting the involvement of the identified mutation in the FA phenotype. The second ROH is located in chromosome 10 and includes the *ERCC6* gene. By filtering pathogenic variants from WES data, we identified a novel missense mutation c.[4114G>A];[4114G>A]: p.[Gly1372Arg];[Gly1372Arg] at homozygous state occurring in exon 21 of *ERCC6* which is the major gene underlying cerebro‐oculo‐facio‐skeletal (COFS) syndrome. By analyzing 37 genes reported as being involved in DSD (Kyriakou, Lucas‐Herald, McGowan, Tobias, & Ahmed, 2015) and the genes they interact with, a novel compound heterozygous mutation in *EFCAB6* gene, a nonsense and a missense mutations c.[322C>T];[4489C>T]: p.[Arg108*];[Arg1497Trp] affecting respectively exon 4 and exon 32 were identified. Sanger sequencing confirmed the segregation of all the variants identified above by the analysis of parents samples (Figure 4). In fact, the nonsense mutation has been inherited from the mother while the missense mutation has been inherited from the father. Thus, these results confirmed that the two compound heterozygous variants are in trans.

## 4 DISCUSSION

We report a rare case of comorbidity involving three rare autosomal recessive diseases namely, FA, DSD, and COFS, affecting the same individual. Genetic analysis, using homozygosity mapping and WES, showed that patient S156‐V‐2 is carrying a nonsense mutation, p.[Arg798*];[Arg798*] in *BRIP1/FANCJ* gene, causing FA, a compound heterozygous mutation in *EFCAB6* gene, p.[Arg108*];[Arg1497Trp] causing DSD and a missense mutation, p.[Gly1372Arg];[Gly1372Arg] in *ERCC6* gene responsible for COFS syndrome.

BRIP1 (BRCA1 Interacting Protein C‐Terminal Helicase 1), is a Fanconi anemia gene (*FANCJ)* (Litman et al., 2005) that functions in DNA damage repair (Bridge, Vandenberg, Franklin, & Hiom, 2005; Litman et al., 2005).

Homozygous mutations in *BRIP1/FANCJ* result in FA phenotype (Kumaraswamy & Shiekhattar, 2007), while heterozygous truncating mutations are associated with a moderate risk of breast cancer (Levran et al., 2005; Seal et al., 2006; Walsh & King, 2007).

The most widespread pathogenic mutation identified in *BRIP1/FANCJ* is p.[Arg798*]. This mutation has been initially described in 2005 to be associated with a lack of BRIP1 expression and hence a deficiency of double‐stranded DNA breaks repair by homologous recombination (Levitus et al., 2005; Levran et al., 2005; Litman et al., 2005). This truncating mutation has been found in patients presenting a severe aplastic anemia from diverse populations, suggesting that it is either a relatively ancient founder mutation or a recurrent one (Levitus et al., 2005; Seal et al., 2006). The p.[Arg798*] mutation is common in Saudi FA patients(40% of all FA patients),compared to only 2% among FA European patients (Ghazwani et al., 2016; Levitus et al., 2005). It is notable that our patient presents more severe skeletal deformities than all previously described patients having this mutation, suggesting that they might result from the mutation identified in the *ERCC6* gene (ERCC excision repair 6, chromatin remodeling factor) which is involved in transcription‐coupled excision repair (Meira et al., 2000). All mutations identified in this gene are associated with Cockayne syndrome type B and COFS syndrome. COFS is a rare autosomal recessive disorder characterized by microcephaly, cataract, microphthalmia, distinctive facial dysmorphism and arthrogryposis (Lowry, MacLean, McLean, & Tischler, 1971; Preus & Fraser, 1974). This disorder is genetically heterogeneous and, at present, four different genes called excision repair cross‐complementing genes (*ERCC6, ERCC2, ERCC5,* and *ERCC1*) are responsible for this phenotype (Jaakkola et al., 2010). Here, we report a novel mutation in *ERCC6* gene that might explain all or at least a part of the facial dysmorphism and skeletal malformations observed in our patient. Interestingly, such complex overlapping features including FA and other DNA‐repair disorders were described in a recent study that reported a malfunction of the nuclease *ERCC1*‐*ERCC4* (*XPF*) resulting in complex clinical manifestations comprising *Xeroderma pigmentosum *(XP), Cockayne syndrome (CS), and Fanconi anemia (FA) (Kashiyama et al., 2013). In another study, mutations in *ERCC4* were described to be involved in FA but without the features of CS and XP (Bogliolo et al., 2013). Before Kashiyama's report of the *ERCC1* mutant patient with COFS syndrome, Jaspers et al. (2007) had reported a first case of *ERCC1* F231L mutation with mild NER deficiency, heterogeneous congenital COFS syndrome, and severe pre‐ and postnatal growth failure. This was followed by characterization of the loss of *ERCC1‐XPF* optimal interaction by Faridounnia et al. (2015). COFS syndrome has been mainly reported to be linked to *CSB*, *XPD*, or *XPG* mutations (Drury et al., 2014; Laugel et al., 2010; Nouspikel, 2009; Powell, Meira, & Friedberg, 2000). Taken together, these findings demonstrate that defects occur in both of *ERCC4* and *ERCC1* can result in either CS or the combined XP‐CS‐FA phenotype (Kashiyama et al., 2013).In this regard, the question that arises is that whether the severe skeletal abnormalities result from the *FANCJ* or *ERCC6* mutation. More pronounced skeletal deformities observed in our patient supports the involvement of the *ERCC6* mutation. Furthermore, the predicted result from in silico tools (SIFT = 0.005, Varsome = 0.9994, MutationTaster = 1) showed that the pathogenic effect for the *ERCC6* mutation is the second hypothesis.

Despite being consanguineous, the patient S156‐V‐2 has two novel deleterious mutations at compound heterozygous state that are responsible for sex reversion. DSD diseases are known to be clinically very heterogeneous. Thus, a careful neonatal exam is crucial (Lee, Houk, Ahmed, & Hughes, 2006). In our case study, the inspected DSD was detected by karyotype analysis and confirmed by WES.

Absence or reduced expression of the *EFCAB6* gene, that encodes DJBP, leads to a decrease in the Androgen receptor (AR) transcription activity (Niki, Takahashi‐Niki, Taira, Iguchi‐Ariga, & Ariga, 2003). It has been suggested that DJBP plays a role in spermatogenesis or fertilization (Niki et al., 2003). Besides, the DJBP protein is a part of more than 70 different binding proteins that interact with AR for its downstream action (Gottlieb, Beitel, Nadarajah, Paliouras, & Trifiro, 2012). Indeed, defects in the androgen receptor gene in 46,XY individuals affect the androgen‐dependent male sexual development, leading to an androgenin sensitivity syndrome (AIS) (Quigley et al., 1995; Sultan et al., 2002).The resulting phenotype exhibit sa genetic makeup of a male with the physical traits of a female, as observed in the present case.

The co‐occurrence of more than one genetic disease affecting one individual and/or different members from the same family is known as comorbidity. This phenomenon seems to be frequently reported in highly inbred populations as described by Romdhaneet al. (2016), where 75 disease associations were reported. While Libyan population is characterized by its heterogeneous ethnic background and high rate of consanguinity (Abudejaja et al., 1987), the report of multiple disease associations in the same individual could be expected. The main challenging consequence of comorbidity is undoubtedly the possibility of misdiagnosis especially when phenotypes are extremely rare and overlapping as one phenotype could be hidden by another (Romdhaneet al., 2016).

**Figure 5:**
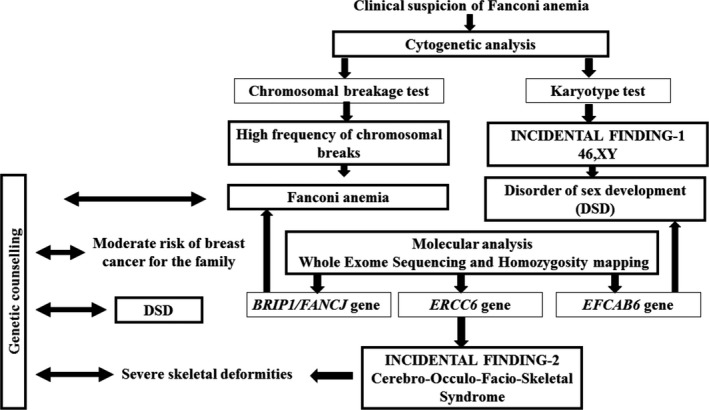
Strategy of cytogenetic and molecular investigation

The initial diagnosis based on clinical and cytogenetic findings for FA and DSD was considered as an important starting point for further molecular approaches in order to characterize each disease (Figure 5). During the last decade, next‐generation sequencing and particularly WES has become an undeniable and a valuable tool for the identification of genes underlying rare monogenic diseases and in case of comorbidity as described here (Cullinane et al., 2011; Chong al., 2015; Gilissen, Hoischen, Brunner, & Veltman, 2012; Margolin et al., 2013). Targeted gene sequencing could be an alternative but it will not identify mutations in hitherto unknown genes. Multiple strategies have been used to prioritize WES variants including homozygosity mapping which was successful in the partial elucidation of the molecular etiology of our patient's phenotype (Becker et al., 2011; Erlich et al., 2011; Gilissen et al., 2012). The autozygosity analysis revealed the causative mutations for FA and COFS phenotypes, whereas it has not been effective to characterize the genetic basis of DSD traits. A candidate gene strategy for variant filtering was adopted to identify the compound heterozygous mutations in the *EFCAB6* gene. This highlights that the allelic homogeneity is not always the rule even in highly inbred population (Romdhane & Abdelhak, 2011).The challenge for clinical diagnostic laboratory is no longer a problem of detection, but of interpretation and analysis of the huge amount of generated variants in the context of the patient's phenotype. This is well‐illustrated through the present case. Indeed, the presence of the *BRIP1* mutation is very challenging for genetic counsellors as they should take into consideration not only the FA risk for the descendants but also the breast cancer risk for the ascendants.

In conclusion, the present study represents the first case with the combination of three rare entities: Fanconi anemia (FA) associated with disorders of sex development (DSD) and cerebro‐oculo‐facial‐skeletal syndrome (COFS). It also illustrates that using a combined approach, homozygosity mapping and WES represent a rapid and powerful tool that allows us to reveal pathogenic mutations in autosomal recessive inherited diseases, especially in consanguineous families. Thus, the output is considered as a support for clinical and genetic characterization of comorbid phenotypes and then contributes to a better and accurate health‐care management.

## CONFLICT OF INTEREST

Abir Ben Haj Ali, Ahlem Amouri, Marwa Sayeb, Saloua Makni, Wajih Hammami, Chokri Naouali, Hamza Dallali, Lilia Romdhane, Anu Bashamboo, Kenneth Mcelreavey, Sonia Abdelhak, Olfa Messaoud declare that they have no conflict of interest.

## AUTHOR CONTRIBUTIONS

Abir Ben Haj Ali, Wajih Hammami carried out the experimental work. Abir Ben Haj Al drafted the manuscript. Abir Ben Haj Ali, Marwa Sayeb, Chokri Naouali, Hamza Dallali, Lilia Romdhane, Anu Bashamboo, Kenneth Mcelreavey, Olfa Messaoud analyzed the data. Ahlem Amouri, Saloua Makni, Sonia Abdelhak, Olfa Messaoud planned the experiments. Sonia Abdelhak, Olfa Messaoud finalized the manuscript.
